# STAT3-survivin signaling mediates a poor response to radiotherapy in HER2-positive breast cancers

**DOI:** 10.18632/oncotarget.6855

**Published:** 2016-01-09

**Authors:** Jae-Sung Kim, Hyun-Ah Kim, Min-Ki Seong, Hyesil Seol, Jeong Su Oh, Eun-Kyu Kim, Jong Wook Chang, Sang-Gu Hwang, Woo Chul Noh

**Affiliations:** ^1^ Division of Radiation Cancer Research, Korea Institute of Radiological and Medical Sciences, Seoul, Korea; ^2^ Department of Surgery, Korea Cancer Center Hospital, Korea Institute of Radiological and Medical Sciences, Seoul, Korea; ^3^ Department of Pathology, Korea Cancer Center Hospital, Korea Institute of Radiological and Medical Sciences, Seoul, Korea; ^4^ Department of Genetic Engineering, Sungkyunkwan University, Suwon, Korea; ^5^ Department of Surgery, Breast Cancer Center, Seoul National University Bundang Hospital, Seoul National University College of Medicine, Gyeonggi-do, Korea; ^6^ Stem Cell Regenerative Medicine Center, Research Institute for Future Medicine, Samsung Medical Center, Seoul, Korea

**Keywords:** breast cancer, radioresistance, HER2, STAT3, survivin

## Abstract

Although radiotherapy resistance is associated with locoregional recurrence and distant metastasis in breast cancers, clinically relevant molecular markers and critical signaling pathways of radioresistant breast cancer are yet to be defined. Herein, we show that HER2-STAT3-survivin regulation is associated with radiotherapy resistance in HER2-positive breast cancers. Depletion of HER2 by siRNA sensitized HER2-positive breast cancer cells to irradiation by decreasing STAT3 activity and survivin, a STAT3 target gene, expression in HER2-positive breast cancer cells. Furthermore, inhibition of STAT3 activation or depletion of survivin also sensitized HER2-positive breast cancer cells to irradiation, suggesting that the HER2-STAT3-survivin axis is a key pathway in radiotherapy resistance of HER2-positive breast cancer cells. In addition, our clinical analysis demonstrated the association between HER2-positive breast cancers and radiotherapy resistance. Notably, we found that increased expression of phosphorylated STAT3, STAT3, and survivin correlated with a poor response to radiotherapy in HER2-positive breast cancer tissues. These findings suggest that the HER2-STAT3-survivin axis might serve as a predictive marker and therapeutic target to overcome radiotherapy resistance in HER2-positive breast cancers.

## INTRODUCTION

Breast cancer is the most common cancer in women worldwide, and its incidence continues to rise [1, 2]. Radiotherapy is recommended for most patients for local control following breast conserving surgery, as well as following mastectomy in patients who are at high risk of recurrence [3–5]. However, some patients are resistant to radiotherapy and the failure of local control in breast cancer decreases the overall survival rate of patients [6, 7]. Thus, identifying both a molecular signature to predict the outcome of radiotherapy and targets to sensitize radioresistant cells is essential for improving the efficacy of radiotherapy in breast cancer. Accumulating evidence suggests that differences might exist in the radiation susceptibility of each molecular subtype of breast cancer [4].

Human epidermal growth factor receptor 2 (HER2) is overexpressed in approximately 25–30% of breast cancer patients, and it plays a key role in both the progression and metastasis of breast cancer [8]. High levels of HER2 are associated with poor prognosis and reduced survival rates [9]. Therefore, HER2 inhibition might be an effective strategy to reduce tumor aggressiveness [10, 11]. Several reports have shown that HER2 inhibition sensitizes breast cancer cells to irradiation both *in vitro* and *in vivo* [12–15]. This suggests that HER2 might be a predictive biomarker as well as a molecular target for radiotherapy in breast cancer patients [16]. However, HER-2 status alone cannot be used a predictive marker for survival after postmastectomy radiotherapy [17]. Therefore, the correlation between the molecular profile of breast cancers such as, HER2 and hormone receptor (HR) status, and their susceptibility to radiotherapy needs to be evaluated.

Signal transducer and activator of transcription 3 (STAT3) is a transcription factor that transduces oncogenic signals from cytokines and growth factors to the nucleus [18]. Constitutive activation of STAT3 is frequently observed in a variety of human cancers, including breast cancer [19, 20], and plays a role in tumor progression and resistance to anti-cancer treatments by regulating the growth and survival of tumor cells [18]. In addition, a number of recent studies have shown that STAT3 might be a promising target for treatment of chemo- and radio-resistant tumors [15, 21–24]. Further, increased activation of STAT3 and its target genes, such as survivin, is often associated with tumor resistance to chemotherapy and radiotherapy in the brain, breast, colon, rectum, head, neck, and lung [21, 25]. Inhibition of the STAT3 pathway often sensitizes radio-resistant tumor cells in various cancers to irradiation [15, 21, 22]. Thus, understanding STAT3 signaling is crucial for predicting and overcoming tumor resistance.

In the present study, we investigated the association between breast cancer subtypes and susceptibility to radiotherapy. Our data shows that the HR−/HER2+ subtype of breast cancer is resistant to radiotherapy, and that this radio-resistant phenotype is mediated by HER2-STAT3-survivin signaling. This suggests that targeting HER2-STAT3-survivin signaling might be an effective strategy for adjuvant radiotherapy in the HER2-positive subtype of breast cancer.

## RESULTS

### HER2-positive breast cancer is associated with radiotherapy resistance

**Figure 1:**
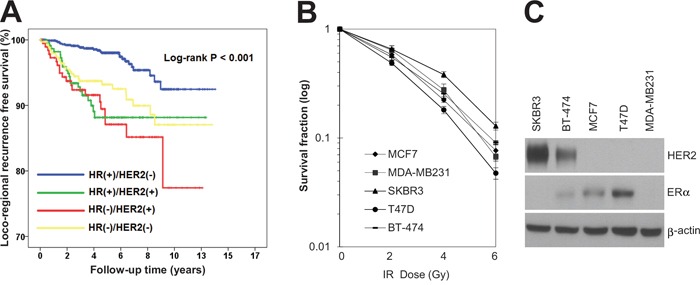
HR−/HER2+ subtype was associated with radioresistance in breast cancer patients as well as breast cancer cell lines **A.** Kaplan–Meier event free survival curve in patients treated with breast-conservation therapy followed by adjuvant radiotherapy (log-rank test, *P* < 0.001). **B.** MCF7, MDA-MB231, SKBR3, T47D, and BT-474 cells were treated with different doses of radiation as indicated. The clonogenic survival fraction was determined by clonogenic assay. **C.** MCF7, MDA-MB231, SKBR3, T47D, and BT-474 cells were analyzed by immunoblotting with anti-HER2 and anti-ER antibodies. β-actin was used as a loading control. The data are presented as the mean ± standard deviation of three independent experiments.

The clinicopathologic features of the patients are summarized in Table 1. Patients were classified into four categories based on the molecular expression of HR (estrogen receptor [ER] and/or progesterone receptor [PR]) and HER2 in their tumors [26, 27]: HR+/HER2−, HR+/HER2+, HR−/HER2+, and HR−/HER2−. The majority of these patients were HR+/HER2− (54.9%, 929 of 1,693 patients), followed by HR−/HER2− (20.5%, 347 of 1,693 patients), HR+/HER2+ (13.6%, 231 of 1,693 patients), and HR−/HER2+ (11.0%, 183 of 1,693 patients). The locoregional recurrence-free survival was significantly different among these groups. HR+/HER2− breast cancer patients showed the highest locoregional recurrence-free survival rate, whereas HR−/HER2+ patients had the lowest locoregional recurrence-free survival rate (*P* < 0.001; Figure 1A). This suggests that different molecular subtypes of breast cancer are inherently associated with different sensitivities to radiotherapy. Therefore, we hypothesized that the HR−/HER2+ subtype is associated with higher radiotherapy resistance compared to other molecular subtypes of breast cancer. To test this possibility, a clonogenic survival analysis in response to various doses of irradiation was performed using various breast cancer cell lines, including MCF7 and T47D (HR+/HER2−), MDA-MB231 (HR−/HER2−), BT474 (HR+/HER2+), and SKBR3 (HR−/HER2+). Interestingly, we observed that the HER2-positive (HR−/HER2+) breast cancer cell line SKBR3 exhibited the most radioresistant phenotype of all breast cancer cells tested (Figure 1B and C). Taken together, our clinical and pre-clinical results suggested that HER2-positive breast cancer is resistant to radiotherapy.

**Table 1:** The clinicopathologic characteristics of patients

Variable	Total (n=1693) No. (%)
Age (yr) at the time of surgery	
<50	1071 (63.3%)
≥50	622 (36.7%)
Breast operation	
Mastectomy	1286 (76.0%)
Breast conserving surgery	407 (24.0%)
T stage	
Tis	132 (7.8%)
T1	944 (55.8%)
T2	503 (29.7%)
T3	61 (3.76%)
T4	50 (3.0%)
Unknown	3 (0.2%)
N stage	
N0	1048 (61.9%)
N1	290 (17.1%)
N2	229 (13.5%)
N3	116 (6.9%)
Unknown	10 (0.6%)
Stage	
0	127 (7.5%)
I	723 (42.7%)
II	464 (27.3%)
III	370 (21.9%)
Unknown	10 (0.6%)
Hormonal receptor	
ER+ and/or PR+	1160 (68.5%)
ER- and PR-	533 (31.5%)
HER2	
Negative	1276 (75.4%)
Positive	417 (24.6%)
Molecular subtype	
HR+/HER2−	929 (54.9%)
HR+/HER2+	231 (13.6%)
HR−/HER2+	186 (11.0%)
HR−/HER2−	347 (20.5%)

ER, estrogen receptor; PR, progesterone receptor; HR, hormone receptor (ER or PR); HER2, human epidermal growth factor receptor 2

### HER2-induced activation of STAT3 signaling leads to radioresistance in HER2-positive breast cancer cells

**Figure 2:**
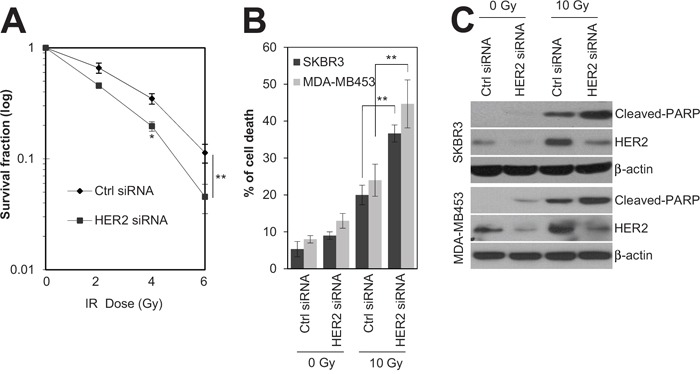
HER2 depletion sensitized HR−/HER2+ breast cancer cells to irradiation **A.** SKBR3 cells were transfected with 100 nM of control siRNA or HER2 siRNA. After 48 h, the cells were treated with different doses of radiation, as indicated. **B** and **C.** SKBR3 cells or MDA-MB453 cells were transfected with 100 nM control siRNA or HER2 siRNA. After 48 h, the SKBR3 cells or MDA-MB453 cells either were left untreated (Ctrl) or treated with 10 Gy of radiation (IR) for 48 h. Cell viability was determined with a FACScan flow cytometer, and data are presented as the percentage of propidium iodide-positive cells (B). The cells were analyzed by immunoblotting with anti-cleaved-PARP and anti-HER2 antibodies. β-actin was used as a loading control (C). The data represent typical results and are presented as the mean ± standard deviation of three independent experiments; *P < 0.05 or **P < 0.01 compared with irradiated siRNA control cells (A and B).

**Figure 3:**
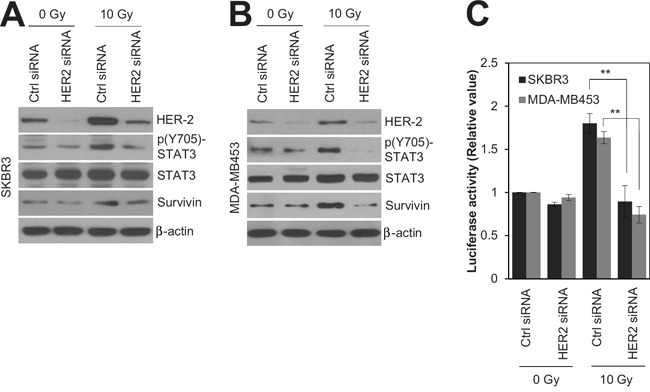
HER2 depletion radiosensitized HR−/HER2+ breast cancer cells by modulating STAT3 activity SKBR3 cells or MDA-MB453 cells were transfected with 100 nM control siRNA or HER2 siRNA. After 48 h, SKBR3 cells or MDA-MB453 cells were either left untreated (Ctrl) or treated with 10 Gy of radiation (IR) for 48 h. **A** and **B.** Cells were analyzed by immunoblotting with the indicated antibodies. β-actin was used as a loading control. **C.** STAT3 activity in each sample was determined by a STAT3 activity assay. The data represent typical results and are presented as the mean ± standard deviation of three independent experiments; **P < 0.01 compared with irradiated siRNA control cells (C).

Since HER2 expression is associated with radioresistance in breast cancer [4, 16], siRNA-mediated silencing of HER2 was employed to test whether HER2 is the key mediator of radioresistance in HER2-positive breast cancer cells. As expected, silencing of HER2 by siRNA significantly decreased the survival of SKBR3 cells in response to various doses of radiation (Figure 2A). In addition, we observed that HER2 depletion led to an increase in radiation-induced cell death in HER2-positive SKBR3 and MDA-MB453 breast cancer cells [28, 29] (Figure 2B and C). This suggests that HER2 is the major regulator of radioresistance in HER2-positive breast cancer cells. Next, we examined the signaling pathways involved in HER2-mediated radioresistance of breast cancer cells. Among several oncogenic pathways, we found that HER2 promotes radiation-induced activation of STAT3, one of the key signaling molecules in multiple radioresistant cancers [21]. Radiation-induced activation of STAT3 was inhibited by HER2 depletion in HER2-positive SKBR3 and MDA-MB453 breast cancer cells (Figure 3A and B), as determined by the decreased level of phosphorylated STAT3 and survivin [18]. Furthermore, the direct transcriptional activity of STAT3 was measured using a STAT3 reporter plasmid, which has the STAT3-binding element for luciferase expression [30]. Data from the luciferase reporter assay also indicated that HER2 depletion inhibits radiation-induced STAT3 activation in irradiated cells. (Figure 3C). Taken together, these results suggested that HER2 enhances radioresistance by activating STAT3 signaling in HER2-positive breast cancer cells.

### Inhibition of the HER2-STAT3-survivin axis increases radiation sensitivity in HER2-positive breast cancer cells

**Figure 4:**
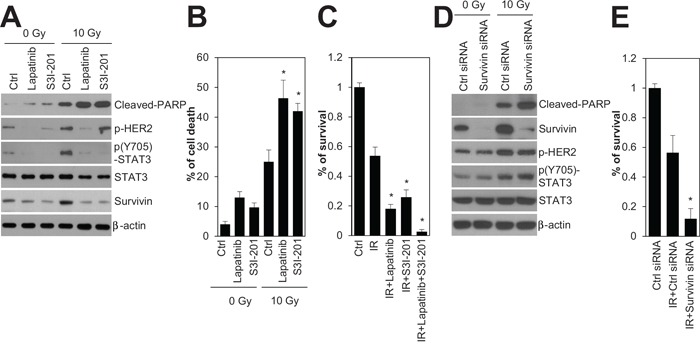
Inhibition of HER2, STAT3, and survivin radiosensitized HR−/HER2+ SKBR3 breast cancer cells **A** and **B.** SKBR3 cells were untreated (Ctrl) or treated with 10 Gy of radiation (IR) in the absence (Ctrl; DMSO) or presence of 1 μM lapatinib or 100 μM S3I-201, and then incubated for 24 h. Cell viability was determined with a FACScan flow cytometer and data are presented as percentage of propidium iodide-positive cells (B). **C.** SKBR3 cells untreated (Ctrl) or treated with 3 Gy of radiation (IR) in the absence (Ctrl; DMSO) or presence of 1 μM lapatinib, 100 μM S3I-201, or 1 μM lapatinib plus 100 μM S3I-201. **D** and **E.** SKBR3 cells were transfected with 100 nM control siRNA or survivin siRNA. After 48 h, the cells were treated with 10 Gy (D) or 3 Gy of radiation (E). The cells were analyzed by immunoblotting with the indicated antibodies. β-actin was used as a loading control (A and D). Clonogenic survival was determined by colony formation assay. Colony formation was quantified by automatic colony counter (C and E). The data represent typical results and are presented as the mean ± standard deviation of four independent experiments; **P < 0.01 compared with irradiated control cells (B, C, and E).

To investigate whether inhibition of HER2 and STAT3 sensitizes HER2-positive SKBR3 breast cancer cells to irradiation, we used chemical inhibitors, lapatinib and S3I-201, that target HER2 and STAT3, respectively. Treatment with lapatinib and S3I-201 increased radiation-induced cell death and decreased both radiation-induced STAT3 phosphorylation and survivin expression in HER2-positive SKBR3 breast cancer cells (Figure 4A and B). In the survival analysis, the rate of colony formation in response to 3 Gy irradiation indicated that treatment with lapatinib or S3I-201, when combined with radiation, led to a significant reduction in the survival rate of HER2-positive SKBR3 breast cancer cells (Figure 4C). Next, we examined whether survivin inhibition with siRNA enhanced the radiation sensitivity of HER2-positive SKBR3 breast cancer cells. Similar to the effects of HER2 and STAT3 inhibition, survivin depletion increased radiation-induced cell death and reduced clonogenic survival of HER2-positive SKBR3 breast cancer cells (Figure 4D and E), but it did not affect HER2 and STAT3 phosphorylation (Figure 4D). These results suggested that HER2-STAT3-survivin signaling is a key factor in the radioresistance of HER2-positive breast cancer cells, implying that the HER2-STAT3-survivin axis could be a potential target for adjuvant radiotherapy in HER2-positive breast cancers.

### *In vivo* evidence for a positive correlation between the HER2-STAT3-survivin axis and radiotherapy resistance in HER2-positive breast cancer tissues

**Figure 5:**
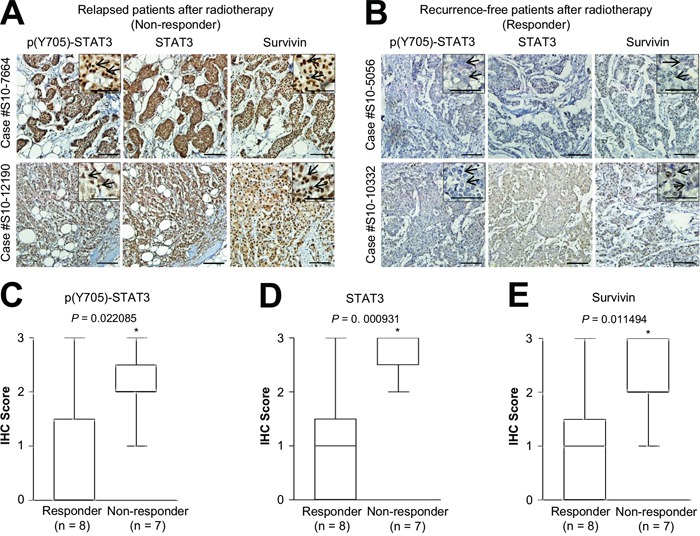
Positive correlation between phosphorylated STAT3, STAT3, and survivin expression and relapsed HER2-positive breast cancer after radiotherapy **A** and **B.** Representative microscopic images of relapsed (A) or recurrence-free (B) HER2-positive breast cancer tissues stained with anti-phosphorylated STAT3 (Y705) (left panel), anti-STAT3 (middle panel), or anti-survivin antibody (right panel). Representative high-magnification images of relapsed (A) or recurrence-free (B) HER2-positive breast cancer tissues (upper right panel). Arrows indicate the nuclear staining pattern of the specific protein. Scale bar, 50 μm. **C-E.** Quantification of phosphorylated STAT3 (C), STAT3 (D), or survivin (D) staining intensities in recurrence-free (responder; *n* = 8) and relapsed (non-responder; *n* = 7) breast cancer tissues. Data are represented by box-and-whisker plots. Staining intensity was scored as follows: 0, no staining; +1, weak; +2, moderate; and +3, strong. *P < 0.05 compared with responder group.

To further examine the physiological relevance of HER2-STAT3-survivin regulation in radiotherapy resistance of HER2-positive breast cancers, we evaluated the expression level of phosphorylated STAT3 (Tyr705), STAT3, and survivin in relapsed (non-responder group; *n* = 7) or recurrence-free (responder group; *n* = 8) HER2-positive breast cancer patients after radiotherapy. Interestingly, we observed that the staining patterns of phosphorylated STAT3, STAT3, and survivin were similar in the serial sections of relapsed HER2-positive breast cancer patients, but not in the recurrence-free patients (Figure 5A and B). In addition, the strong nuclear staining patterns of phosphorylated STAT3 and survivin, indicative of STAT3 activation, were detected in relapsed HER2-positive breast cancer patients rather than in the recurrence-free patients (Figure 5A and B, right upper panels). Further, we found that increased expression of phosphorylated STAT3, STAT3, and survivin was positively associated with the group that was non-responsive to radiotherapy (Figure 5C–E). These observations provided *in vivo* evidence that the HER2-STAT3-survivin axis might confer radiotherapy resistance in HER2-positive breast cancers.

## DISCUSSION

This study provides clinical and experimental evidence for the role of the HER2-STAT3-survivin axis in radiotherapy resistance of HER2-positive breast cancers. We have shown that the HR−/HER2+ subtype of breast cancer is associated with radiotherapy resistance via HER2-mediated regulation of STAT3-survivin signaling. Further, our data suggests that increased HER2-STAT3-survivin expression might confer poor outcomes in response to radiotherapy in patients with HER2-positive breast cancer.

Our clinical data showed that each molecular subtype of breast cancer is associated with different locoregional recurrence rates in patients treated by curative surgery followed by adjuvant radiotherapy. Our observations are similar to those of several other groups that have shown an association between the molecular subtype of breast cancer and increased risk of local or regional recurrence following radiotherapy [4, 9] [17]. For instance, postmastectomy radiotherapy confers survival benefits in the HR+/HER2- subtype of breast cancer. In contrast, HR−/HER2+ tumors are associated with an increased probability of local recurrence and distant metastasis after radiotherapy [17]. Further, many *in vitro* studies have shown that HER2 overexpression or inhibition modulates radiation resistance in breast cancer cells [12–15]. These observations, taken collectively, indicate that the HR−/HER2+ subtype of breast cancer is associated with radiotherapy resistance.

Our data showed that HER2 promotes radioresistance via STAT3-survivin regulation in HER2-positive breast cancers. Similar to our data, recent reports have suggested that STAT3 could be the key downstream mediator of HER2 signaling [31]. Duru et al. have shown that HER2-STAT3 cross-talk increases the aggressiveness and radioresistance of breast cancer stem cells [15]. Chung et al. reported that STAT3 activation by HER2 overexpression promotes cancer stem cell traits that correlate phenotypically with tumor resistance in HER2-expressing breast cancers [32]. Moreover, it has been suggested that downstream pathways of HER2, such as PI3K/Akt and NF-kB, crosstalk with STAT3 signaling in resistant phenotypes of breast cancer [31]. These findings suggest that HER2-STAT3 regulation is crucial regulator for tumor radioresistance of HER2-positive breast cancers.

Survivin plays a key role in the inhibition of apoptosis and promotion of mitosis in response to anticancer therapies [25, 33]. A previous study suggested that STAT3 activation is correlated with survivin expression in high-risk breast cancer patients [19]. Our present study showed that survivin is a downstream effecter of HER2-STAT3 regulation in response to irradiation of HER2-positive breast cancer cells, and that increased STAT3-survivin expression was associated with a poor response to radiotherapy in HER2-positive breast cancers, suggesting that STAT3-survivin is a potential biomarker for radioresistance in HER2-positive breast cancers. Regarding the role of survivin in radioresistance, survivin inhibits apoptosis of tumor cells by directly or indirectly regulating caspase-3/-7 or apoptosis-regulatory factors such as HSP90 and AIF [25]. It also has the ability to promote mitosis as a regulator [34] or DNA repair via Ku70 [25]. Thus, increased survivin by HER2-STAT3 regulation might protect tumor cells from ionizing radiation through multiple mechanisms, such as inhibiting apoptosis, promoting mitosis, and enhancing DNA repair in radioresistant HER2-positive breast cancers.

On the basis of pre-clinic evidence indicating that inhibition of HER2 increases radiation sensitivity of breast cancers [13–15, 35], a combination treatment with radiation and HER2 inhibitors (trastuzumab or lapatinib) is currently under investigation in clinical trials [36]. Similarly, our pre-clinical evidence that inhibition of the HER2-STAT3-survivin axis increases radiation sensitivity of HER2-positive breast cancers suggests that targeting the HER2-STAT3-survivin axis may be important to overcoming radiotherapy resistance in HER2-positive breast cancers. Related to this observation, previous work by our group as well as others has shown that STAT3 inhibition is crucial for the treatment of various radioresistant tumors [21, 22, 24]. Further, Li et al. recently reported that trastuzumab resistance is regulated by STAT3-dependent feedback activation in HER2-positive breast and gastric cancers [24]. Therefore, STAT3 activation is a key pathway for the survival of various resistant tumors, including breast cancer.

In conclusion, this study showed that HER2-STAT3-survivin regulation potentiated radiation resistance of HER2-positive breast cancer cells. Our work provides evidence that the HER2-STAT3-survivin axis is a predictive marker and a potential therapeutic target for radiotherapy resistance in HER2-positive breast cancers.

## MATERIALS AND METHODS

### Cell lines and treatments

Human breast cancer cell lines MCF7, MDA-MB231, SKBR3, T47D, and BT474 were purchased from the American Type Culture Collection (Manassas, VA) and grown in Dulbecco's modified Eagle's medium supplemented with 10% fetal bovine serum (HyClone, South Logan, UT) and penicillin/streptomycin at 37°C in a humidified 5% CO_2_ atmosphere. The cells were irradiated using a ^137^cesium (Cs) ray source (Atomic Energy of Canada Ltd., Mississauga, Canada) at a dose rate of 3.81 Gy/min. S3I-201 (100 μM; EMD Millipore, Billerica, MA) and lapatinib (1 μM; Selleckchem, Houston, TX) were used to inhibit STAT3 and HER2 activity, respectively.

### Clonogenic assay

Cell survival after irradiation was determined by a clonogenic assay as described previously [37]. Briefly, various densities of cells treated with different doses of radiation were seeded in triplicate in 60-mm tissue culture dishes. After 10–14 days the colonies were fixed with methanol and stained with a Trypan blue solution. Only colonies containing more than 50 cells using a colony counter (Image Products, Chantilly, VA) were counted as surviving colonies.

### RNA interference

siRNAs were synthesized at Genolution Pharmaceuticals Inc. (Seoul, Korea). The following sequences were used for RNA interference: HER2, 5′-CUGGUGUAUGCAGAUUGCC-3′ and survivin, 5′-AAGGAGAUCAACAUUUUCA-3′. A non-targeting siRNA (Genolution Pharmaceuticals Inc.) was used as a negative control. Transfection of siRNA was performed using G-Fectin (Genolution Pharmaceuticals Inc.) according to the manufacturer's protocol.

### Western blot analysis

Western blotting was performed as described previously [37, 38]. Briefly, proteins were separated by SDS-PAGE and transferred to a nitrocellulose membrane, followed by detection using specific antibodies. The antibodies were used included rabbit monoclonal anti-survivin, rabbit polyclonal anti-phospho-STAT3 (Tyr705), and anti-cleaved-PARP (Asp214) from Cell Signaling Technology (Beverly, MA); mouse monoclonal anti-STAT3, rabbit polyclonal anti-ERa, and anti-HER2 from Santa Cruz Biotechnology Inc. (Santa Cruz, CA); and mouse monoclonal anti-β-actin from Sigma (St. Louis, MO). Blots were developed using horseradish peroxidase-conjugated secondary antibody and an enhanced chemiluminescence detection system (Amersham Life Science, Piscataway, NJ).

### STAT3 activity assay

STAT3 activity was determined as described previously [30]. Briefly, the cells were co-transfected with 21pSTAT3-TA-Luc and control siRNA or HER2 siRNA for 48 h using Lipofectamine 2000 (Invitrogen, Carlsbad, CA). This was followed by either irradiation with 10 Gy or no treatment. The cells were harvested after 24 h using a passive lysis buffer, and luciferase activity was evaluated using the Dual Luciferase Reporter Assay Kit (Promega, Madison, WI) on a Wallac Victor2 plate reader (Perkin Elmer Corp., Norwalk, CT).

### Cell death analysis

A cell death analysis was performed as described previously [37]. Briefly, cells were trypsinized and washed, followed by incubation with propidium iodide (5 μg/mL) for 10 min. The cells were analyzed with a FACScan flow cytometer (Becton Dickson, Franklin Lakes, NJ).

### Patient population for locoregional recurrence-free survival analysis

Between January 1980 and September 2010, a total of 1,693 primary breast cancer patients were included in this retrospective analysis. All patients were treated by curative surgery and adjuvant radiotherapy prior to this study. The clinical and pathologic data were obtained from a database of the Breast Cancer Center, Korea Cancer Center Hospital [39].

### Classification of breast cancer patients based on the molecular subtypes of tumors

A pathologist evaluated ER, PR, and HER2 expression in samples from each case by immunohistochemistry (IHC) immediately after surgery. Positive staining for ER or PR was defined as staining of at least 10% of the nuclei in 10 high-power fields, and HER2 positivity was defined by an IHC staining intensity of 3+ or *HER2* gene amplification by fluorescence *in situ* hybridization. Patients were classified into the following four molecular subtypes based on tumor expression of ER, PR, and HER2: (a) HR+/HER2- (ER- and/or PR-positive and HER2-negative), (b) HR+/HER2+ (ER- and/or PR-positive and HER2-positive), (c) HR−/HER2+ (ER-negative, PR-negative, and HER2-positive), and (d) HR−/HER2- (ER-negative, PR-negative, and HER2-negative).

### Immunohistochemistry

The specimens for IHC were obtained from paraffin blocks of HER2 overexpressing primary breast cancer tissue that had been removed by curative surgery. The non-responder group to radiotherapy was defined as the group of patients who showed locoregional recurrence within 1 year after completion of radiotherapy. The responder group was defined as the group of patients who showed no evidence of disease during the follow-up period, which was at least 2 years from the completion of radiotherapy. We matched patients from each group by pathologic TNM staging and HR status. IHC experiments were performed as previously described [40, 41]. Briefly, immunohistochemical staining was performed using an anti-STAT3 mouse monoclonal antibody (1:200 dilution; Santa Cruz), anti-phospho-STAT3 rabbit polyclonal antibody (1:50 dilution; GeneTex, Irvine, CA), or anti-survivin rabbit monoclonal antibody (1:100 dilution; Cell Signaling Technology). Immunostaining was performed using the avidin-biotin-peroxidase method, according to the manufacturer's instructions (Invitrogen). Staining intensity was scored as follows: 0 (no visible staining), 1+ (faint staining), 2+ (moderate staining), and 3+ (strong staining).

### Statistical analysis

For the survival analysis, locoregional recurrence-free survival was defined as the time from the first diagnosis of primary breast cancer to the time of first detection of locoregional recurrence by physical examination or radiological imaging. The Kaplan-Meier method with log-rank test was used for the statistical analysis. A two-tailed Student's *t*-test was performed to analyze statistical differences between groups. *P* < 0.05 was considered statistically significant.
